# Genome wide association study for gray leaf spot resistance in tropical maize core

**DOI:** 10.1371/journal.pone.0199539

**Published:** 2018-06-28

**Authors:** Maurício Carlos Kuki, Carlos Alberto Scapim, Evandrei Santos Rossi, Claudete Aparecida Mangolin, Antônio Teixeira do Amaral Júnior, Ronald José Barth Pinto

**Affiliations:** 1 Departamento de Agronomia, Universidade Estadual de Maringá, Maringá, Paraná, Brasil; 2 Laboratório de Melhoramento Genético Vegetal, Universidade Estadual do Norte Fluminense Darcy Ribeiro, Campos dos Goytacazes, Rio de Janeiro, Brasil; University of Western Sydney, AUSTRALIA

## Abstract

Gray leaf spot is a maize foliar disease with worldwide distribution and can drastically reduce the production in susceptible genotypes. Published works indicate that resistance to gray leaf spot is a complex trait controlled by multiple genes, with additive effect and influenced by environment. The aim of this study was to identify genomic regions, including putative genes, associated with resistance to gray leaf spot under natural conditions of disease occurrence. A genome wide association study was conducted with 355,972 single nucleotide polymorphism markers on a phenotypic data composed by 157 tropical maize inbred lines, evaluated at Maringá –Brazil. Seven single nucleotide polymorphisms were significantly associated with gray leaf spot, some of which were localized to previously reported quantitative trait loci regions. Three gene models linked to the associated single nucleotide polymorphism were expressed at flowering time and tissue related with gray leaf spot infection, explaining a considerable proportion of the phenotypic variance, ranging from 0.34 to 0.38. The gene model GRMZM2G073465 (bin 10.07) encodes a cysteine protease3 protein, gene model GRMZM2G007188 (bin 1.02) expresses a rybosylation factor-like protein and the gene model GRMZM2G476902 (bin 4.08) encodes an armadillo repeat protein. These three proteins are related with plant defense pathway. Once these genes are validated in next studies, they will be useful for marker–assisted selection and can help improve the understanding of maize resistance to gray leaf spot.

## Introduction

Maize is considered the most important cereal in Brazil, with a total cultivated area of 15.9225 million hectares and an average yield of 5,192 kg ha^-1^ in the 2016/17 season; Brazil is considered the third-highest maize producer worldwide, following the United States and China [1]. Management of this crop has drastically changed in the last few decades with the use of improved genotypes, fertilizers, and pest and weed control. However, diseases are becoming an important problem for production, especially in tropical regions [2].

Gray leaf spot (GLS) is a leaf disease caused by the polycyclic pathogens *Cercospora zeae-maydis* and *Cercospora zeina* [3,4]. GLS has a worldwide distribution and can reduce maize production by approximately 40%, mainly in susceptible genotypes [5,6]. With molecular techniques, it was possible to verify that *C*. *zeina* has a major distribution in Brazil and African continent, whereas *C*. *zeae-maydis* is predominant inmost maize producing regions of United States of America [7,8]. One of the main reasons for GLS damage in commercial crops is the increasing use of no-tillage soil, which allows fungal inocula to survive in crop residues and infect crops in early stages of development [7]. GLS symptoms are characterized by the formation of rectangular lesions on leaves during the flowering period, developing from the lower leaves to the upper leaves and reducing the photosynthesis and potential yield of the plants [8].

A more effective and ecologically correct method for controlling GLS is the use of host plant resistance. However, published works indicate that GLS resistance is a complex trait controlled by multiple genes, each one with additive effects [2,9] and strongly influenced by the environment [10]. Many quantitative trait loci (QTLs) closely linked to molecular markers have been mapped in different populations developed from crosses of inbred lines with contrasting levels of resistance [11,12,9], but no QTL reported explains a large percentage of the phenotypic variation. Also, the use of genetic information from recombinant mapping of GLS resistance in marker-assisted selection (MAS) is very limited, as these QTLs are not consistent between the different populations.

The use of single nucleotide polymorphisms (SNPs) identified by the new method of genotyping-by-sequencing (GBS) is a great tool in genome-wide association studies (GWAS) to identify QTLs that explain the phenotypic variation of traits of interest, leading to more effective strategies for the use of molecular markers in crop improvement [13]. Association mapping was successfully used in maize to identify genomic regions that confer resistance for southern leaf blight by *Bipolaris maydis* [14], northern leaf blight by *Exserohilum turcicum* [15], maize head smut by *Sphacelotheca reiliana* [16] and common rust by *Puccinia sorghi* [17].

To date, a genome-wide association studies involving GLS resistance have been reported by [18], where the authors obtained 51 significant SNPs from 161 Chinese inbred lines and identified three candidate genes related to plant defense. However, little is known about GLS resistance in tropical field corn and popcorn, which is an important specialty crop. Thus, the objectives of this study were to *i*) measure phenotypic variation among tropical inbred lines of common maize and popcorn for GLS resistance, *ii*) identify SNPs and putative genes associated with a response to gray leaf spot under natural inoculation, and *iii*) characterize the putative genes by comparing them to genes from previously published studies of GLS resistance.

## Materials and methods

### Plant materials and experimental design

The UEM core maize panel was assembled with a total of 183 tropical inbred lines (98 are field corn and 85 are popcorn genotypes), which have already been genotyped (Table 1). Only 157 genotypes were phenotyped in a field experiment conducted at the Experimental Farm of Iguatemi (FEI)–State University of Maringá (UEM), Maringá city, Brazil localized at the geographical coordinates 23°20’48” S, 52°04’17” W and at 540 m in altitude during the main season of 2016/2017 in a soil classified as Dystrophic Red Argissolo of medium texture. The inbred lines were evaluated in a randomized complete block design, with two replicates. An experimental unit consisted of 6-m-long single rows, 0.9 m apart, with a total of 30 plants per plot. Artificial irrigation and normal agronomic practices of crop development were followed.

**Table 1:** Diverse panel with 183 field corn and popcorn inbred lines.

N^o^	Genotype	N^o^	Genotype	N^o^	Genotype	N^o^	Genotype
1	1-GP1*	47	51-P8-2-MULT *	93	101-AG6018-24H12.2 *	139	156-CML12 *
2	2-GP4*	48	52-T4-DKB747-38H17.2 *	94	102-PREMIUM-28H13.2*	140	157-GP12 *
3	3-P9-4-6*	49	53-P11-2 *	95	103-CML19*	141	159-GP5*
4	4-AG8080-7H3.1*	50	54-GP10 *	96	104-P1-3*	142	160-SPEED-81H33.1*
5	5-T5-AVANT-14H5.5*	51	56-DKB747-36H17.2*	97	105-DKB747-45H17.5*	143	161-P6-11*
6	6-POP103-88.1*	52	57-P7-4-11*	98	106-ANGELA-L70*	144	162-P1780*
7	7-30F33-71H26.2*	53	58-P9-5-1*	99	107-GP11-1*	145	163-POP102-91.2*
8	8-P7-L7-1*	54	59-POP202-177.1*	100	108-FORT-85H6.2–242*	146	164-30-11*
9	10-POP101-201-3*	55	60-FLASH-20H11.1*	101	109-DKB747-29H17.3*	147	165-POP101-197.1*
10	11-DKB350-78H30.1*	56	61-DAS422-80H31.2*	102	110-ANGELA-L65*	148	167-29-154*
11	12-DKB747-50H17.6*	57	62-P8-2-2-5*	103	111-AG8080-8H3.2–6*	149	DAS422-79H31.1*
12	13-BEIJAFLOR-L53*	58	63-AG9090-56H21.1*	104	112-VIÇOSA-L88*	150	DKB74740H17.3*
13	14-P8-1-1*	59	64-P8-1-5-10*	105	115-DKB747-37H17.2*	151	171-FORT-86H6.3*
14	15-P20 *	60	65-P9-1-2*	106	116-BEIJAFLOR-L59*	152	173-A2560-170*
15	16-FORT-87H6.4–248*	61	66-30-23*	107	117-GP14*	153	174-A2560-176*
16	17-P1-9*	62	67-POP201-198.4*	108	119-VIcOSA-L77*	154	175-A2560-164*
17	18-P11-1*	63	68-A2560-66H23.4*	109	120-P15*	155	176-DKB747-41-101*
18	19-P3-3T*	64	69-TORK-54H20.3*	110	121-ANGELA-L71*	156	177-DKB747-47-121*
19	20-T1-P8-2*	65	70-A2560-64H23.2*	111	122-30F33-70H23.1*	157	178-DKB747-48-124*
20	21-STRIKE-67H25.1*	66	71-31-88*	112	124-UFV-L80*	158	179-DAS422-8-222*
21	22-ANGELA-L63*	67	72-P7-4-5*	113	126-30F98-75H29.2*	159	180-DKB747-42-104*
22	23-P8-1-5-4*	68	74-P9-12-1*	114	127-ANGELA-L66*	160	181-AG9090-57-155*
23	24-POP201-195.1*	69	76-POP102-90.1*	115	128-P3-1-2*	161	182-DKB747-44-110*
24	25-30F33-69H26.1*	70	77-29-14*	116	129-P1-8*	162	183-P9-2-3*
25	26-P1-12*	71	78-31-97*	117	130-BEIJAFLOR-L52*	163	185-P9-1-6 *
26	27-GP13*	72	79-POP202-88.2*	118	131-CD303-90H4.3*	164	188DKB35077-H30.1
27	28-P9-1*	73	80-30-15*	119	134-P9-5-3*	165	189DKB440-73H28.1
28	29-P7-2-4*	74	81-T3-P9-3-2*	120	136-TORK-53H20.2*	166	PREMIUM 29H13.3
29	30-DKB747-43H17.4*	75	82-POP201-192.1*	121	137-POP203-56.1*	167	191-A11545-27
30	31-CD303-89H4.2*	76	83-29-92 *	122	138-POP102-166.5*	168	192-DAS2C595-95
31	32-30F98-74H29.1*	77	84-POP203-51.2*	123	139-A2560-62H23.2*	169	193-DAS2C599-93
32	33-P9-4-5*	78	85-29-174*	124	140-POP101-195.2*	170	195-BEIJAFLOR-L55
33	35-VIÇOSA-L75*	79	86-CML13 *	125	141-P9-1-3*	171	197-SE013-289-2
34	36-P6-1*	80	88-POP103-80.5*	126	142-FLASH-22H11.1*	172	198-RS20-257-2
35	37-GP15*	81	89-30-29*	127	144-P9-11-1*	173	199-PRO23-245-1
36	38-AVANT-10H5.1*	82	90-POP103-81.4*	128	145-BEIJAFLOR-L76*	174	200URUG298-982
37	39-DKB747-41H17.3*	83	91-31-124*	129	146-A2560-63H23.2*	175	PA170ROXO324-2
38	40-P8-1-5-9*	84	92-POP202-76.1*	130	147-P8-1-5-5*	176	202-BOYA462-110-2
39	41-P1-19*	85	93-31-33*	131	148-P9-8-1*	177	203-BOZM-260-36-2
40	42-P18*	86	94-P8-2-2-4*	132	149-TORK-55H20.3*	178	BARÃOVIÇOSA1342
41	43-DKB350-76H30.1*	87	95-CML22*	133	150-P7-2-1*	179	CHZM13134-66-2
42	46-P4-4*	88	96-P19*	134	151-FORT-84H6.1*	180	207-SAM274-2
43	47-P8-2-2-2*	89	97-DKB350-19H9.1*	135	152-P8-1-5-13*	181	209-PARA172-76-2
44	48-P12-1*	90	98-AVANT-13H5.4*	136	153-CD303-91.H4.4*	182	ARZM0583-122-2
45	49-AVANT-12H5.3*	91	99-W57*	137	154-GP3*	183	212-CMS-SYN
46	50-P9-7-2*	92	100-P7-2-3*	138	155-DAS2C599-95H34.4*		

*Genotypes evaluated at 2016/17 crop season.

### Phenotypic measurements and statistical analysis

The inbred lines were evaluated for GLS severity, caused by *Cercospora zeina*, under natural conditions of disease infection. Evaluation was performed 25 days after the flowering period, during the kernel milk stage. The disease reaction of each genotype was scored with the scale proposed by [19] with nine levels, which represent the percentage of the infected leaf area (PIFA) as follows: 1–0%; 2–0.5%; 3–10%; 4–30%; 5–50%; 6–70%; 7–80%; 8–90%; and 9–100%.

GLS resistance was evaluated as the average of the plants in a row. An arcsine transformation was performed to correct non-normality of the errors and heterogeneous variances. The mean value for each inbred line was calculated with R software [20] using the lm procedure, considering inbred lines and blocks as fixed effects. The means were estimated with a randomized complete block design model:

*Y*_*ij*_ = *μ* + *G*_*i*_ + *B*_*j*_ + *ε*_*ij*_ where *μ* is the general mean; *G*_*i*_ is the fixed effect of the genotypes, *B*_*j*_ is the fixed effect of the blocks and *ε*_*ij*_ is the random effect of the error, assuming *NID* (0,*σ*).

### Association mapping

The 183 DNA samples were sent to the Institute of Genomic Diversity at Cornell University to undergo genotyping-by-sequencing (GBS), as described by [21]. Reads and tags found in each sequencing result were aligned to the *Zea mays* L. genome reference, version *AGPV3* (B73 RefGen v3 assembly) [22], resulting in a raw dataset of 1,014,070 SNPs. Heterozygosity, alleles with frequencies less than 1% and scaffolds were filtered from the raw data, resulting in 837,522 SNPs in the dataset. The LD KNNI imputation (Linkage disequilibrium k-nearest neighbor imputation) was performed to impute missing data in the data set [23]. SNP markers with a minor allele frequency less than 5% were removed using TASSEL version 5.2 [24] using 60% of the genotypes as the minimum count, resulting in a working dataset of 355,972 SNP markers.

Association analysis between the SNPs and the GLS means was performed with a compressed mixed linear model (MLM) implemented in the GAPIT R package [25]. A kinship coefficient matrix was created using the algorithm proposed by [26] and implemented in the GAPIT R package [25] using the complete set of markers that passed quality filtering as implemented in TASSEL version 5.2 [24]. The number of principal components was selected to estimate the groups in the population based on the Bayesian information criteria (BIC) values and with visual inspection of the number of PCs versus variance decay. The MLM adopted was proposed by [13], with each molecular marker considered a fixed effect and evaluated individually:

*Y* = *X*_*β*_ + *W*_*α*_ + *Q*_*v*_ + *Z*_*u*_ + *ε* where *Y* is the observed vector of means; *β* is the fixed effect vector (*p* x 1) other than molecular markers effects and population structure; *α* is the fixed effect vector of the molecular markers; *ν* is the fixed effect vector from the population structure; *u* is the random effect vector from the polygenic background effect; *X*, *W*, and *Z* are the incidence matrixes from the associated *β*, *α*, *ν*, and *u* parameters; and *ε* is the residual effect vector.

Components of variance for the means of disease severity were estimated with the residual maximum likelihood (REML) method. Broad sense heritability (*h*^2^) was estimated using the formula:

$h^{2}=\frac{\sigma_{g}^{2}}{\sigma_{g}^{2}+\left(\sigma_{e}^{2}/r\right)}$ where $\sigma_{g}^{2}$ is the genotypic variance, $\sigma_{e}^{2}$ is the residual error variance, and *r* is the number of replicates. The false discovery rate (FDR) was used to estimate and control the proportion of false positive results among all discoveries, adjusted to a p-value of 0.05. To determine the amount of variance explained by the top significant SNPs, a linear model was fitted using R software with the lm procedure [20].

To identify gene models for GLS resistance, the physical position of the SNPs were compared with the MaizeGDB database according to version 3 (RefGen_v3) from the reference genome of the maize B73 inbred line, available at the MaizeGDB database. Linkage disequilibrium (LD) between the significant SNPs and the SNPs inside or near the gene models was evaluated using TASSEL version 5.2 [24], with the set of 355,972 SNP markers and a 50-SNP window between sites.

## Results

**Fig 1:**
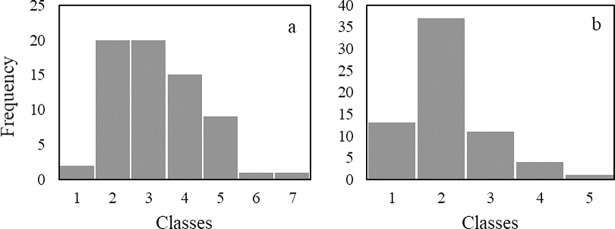
Genotypes frequency among classes, according with scale proposed for Agroceres (1996), for percentage of infected leaf area (PIFA) of gray leaf spot. (a) Popcorn inbred lines and (b) Field corn inbred lines.

The phenotypic means from 157 inbred lines evaluated at Maringá were considered significant (p<0.05) for the PIFA. The average severity of GLS reactions can be considered similar for popcorn and field corn inbred lines, but the popcorn group showed a higher presence of outliers and large mean variations (Fig 1). This result showed that inbred lines differ in GLS resistance between and within genetic groups, which can be important for breeding programs to obtain genetic resistance.

Results from the variance components and broad sense heritability are shown in Table 2. The genetic variation obtained in the experiment was 0.0028, and the residual variance was 0.000276. The broad sense heritability estimate for the PIFA was 0.9 which was very similar to that reported by [18] and [9]. According to [27], traits with higher heritability increase the power of SNP detection in a panel, allowing the identification of true associations between a marker and putative gene.

**Table 2:** Components of variance and heritability to gray leaf spot severity in tropical corn inbred lines.

Variance components	Estimate
$\sigma_{g}^{2}$	0.0028
$\sigma_{e}^{2}$	0.000276
*h*^2^	0.91

**Fig 2:**
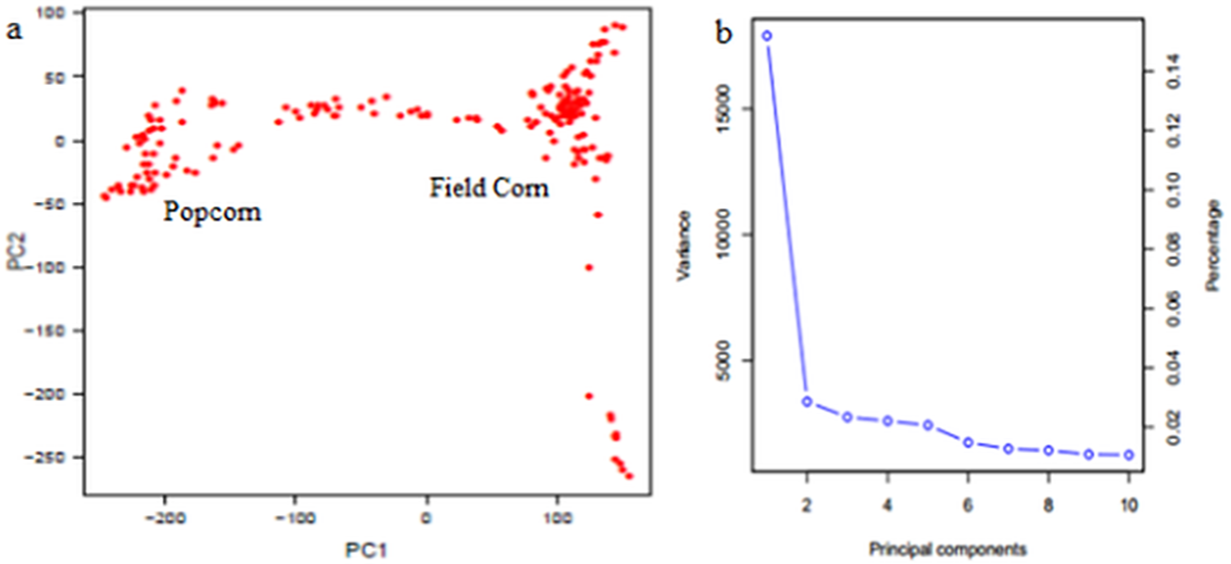
Principal component analysis (PCA) results. a) Graphic plot of the principal component analysis of 183 maize inbred lines, calculated from ~355k SNP markers. The horizontal and vertical axes are the first and second principal components, respectively. b) Eigenvalue accumulation variance among PCs revealed variance contribution to only two PCs.

**Fig 3:**
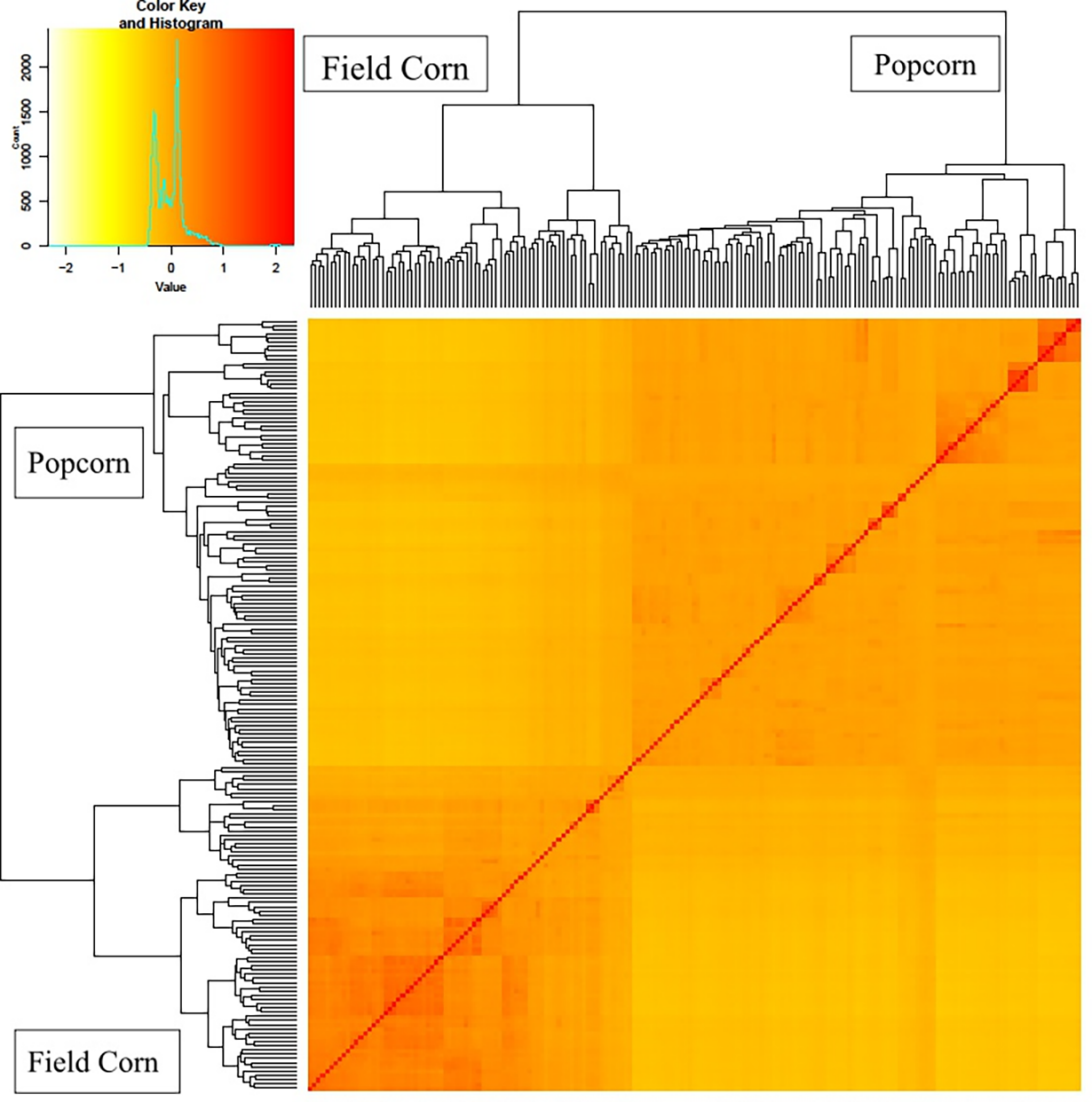
Heatmap of pairwise kinship matrix values, based on a ~355k SNPs on 183 tropical field corn and popcorn, according with VanRaden algorithm. The color histogram shows the distribution of coefficients of coancestry, and the stronger red color indicates the individuals that were more related to each other.

The PCA resulted in two groups based on the eigenvalues of accumulated variances among PCs (Fig 2), which may be related to the presence of a population structure, representing the field corn and popcorn populations in the maize core. A kinship heatmap (Fig 3) showed the clustering between the related genotypes and the formation of the two PCA groups. Tropical maize has a large genetic base compared with that of temperate maize [28], and other subgroups related to the grain type are expected inside the germplasms. Even though the kinship matrix clustered genotypes with *flint* grain together, the number of field corn genotypes was not enough to show different subgroups.

**Fig 4:**
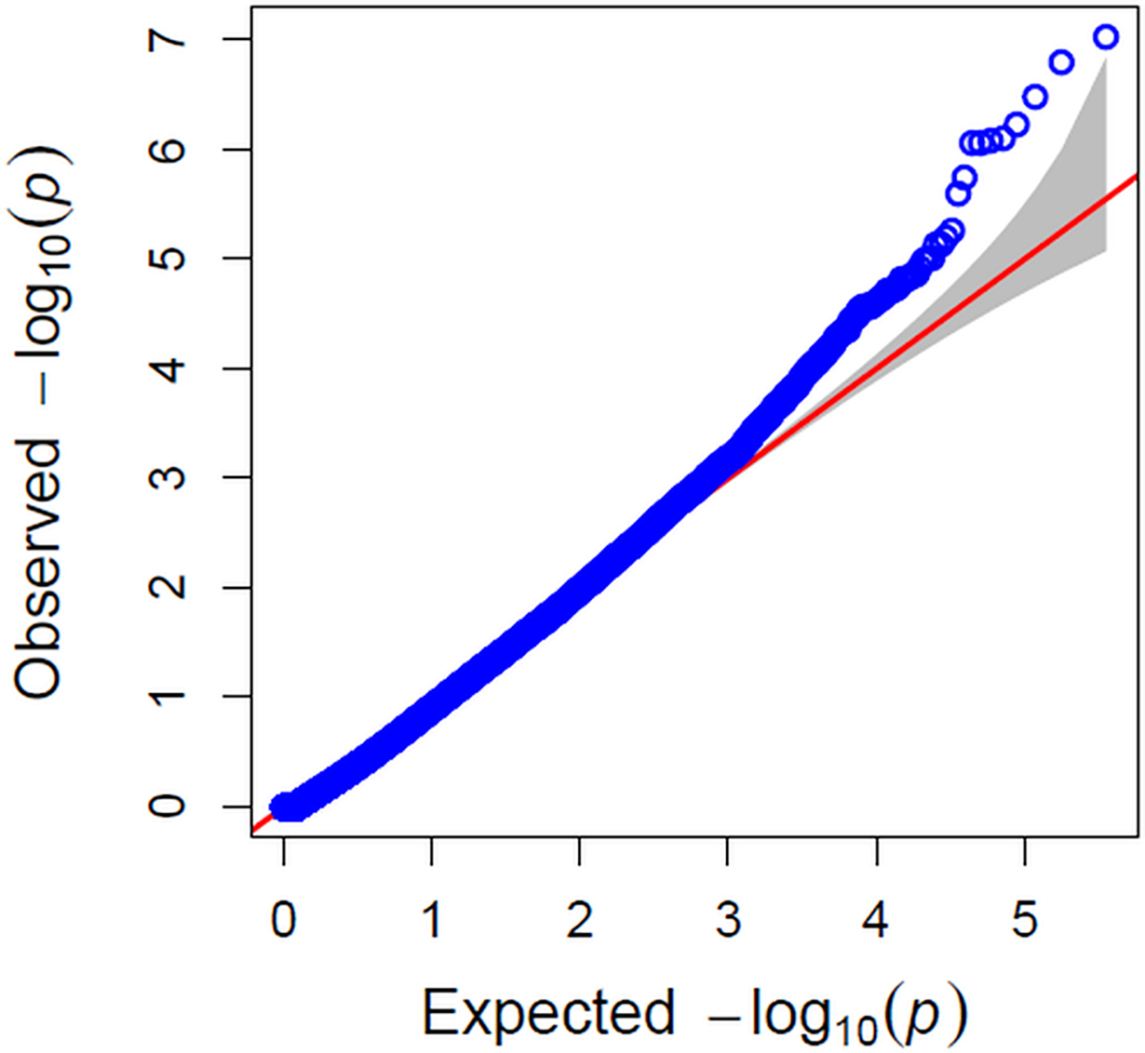
Quantile–quantile plots of estimated −log_10_ (P) from observed vs. expected P values from association analysis. The red line indicates the expected distribution under null hypothesis, and the blue line indicates the distribution from the observed association with the percentage of leaf area infected data.

The results of the marker-trait association for gray leaf spot severity are illustrated in the quantile-quantile plot (QQ-plot–Fig 4). QQ-plots are used for model adjustment because they compare the expected distribution of the association test considering the null hypothesis with the observed SNP results. Any deviation above the diagonal implies differences between the observed and expected values across the genome. The result showed good data adjustment and a few significant SNPs above the interval for the expected values of the null hypothesis.

The results of the association analysis are illustrated in S1 Table. Considering a false discovery rate of 5%, a total of seven SNPs were associated with the severity of gray leaf spot. These SNPs explain a total of 46.68% of the genetic variation for the phenotypic characteristic. Individually, an *R*^*2*^ proportion between 1.48% and 37.50% was explained by these SNPs (S1 Table), which is considered to be similar to the results obtained by [18] with GLS association mapping and with the QTL effects that other studies obtained with recombinant mapping [10,2]. Therefore, these markers can be useful for marker-assisted selection of GLS resistance in breeding programs.

**Fig 5:**
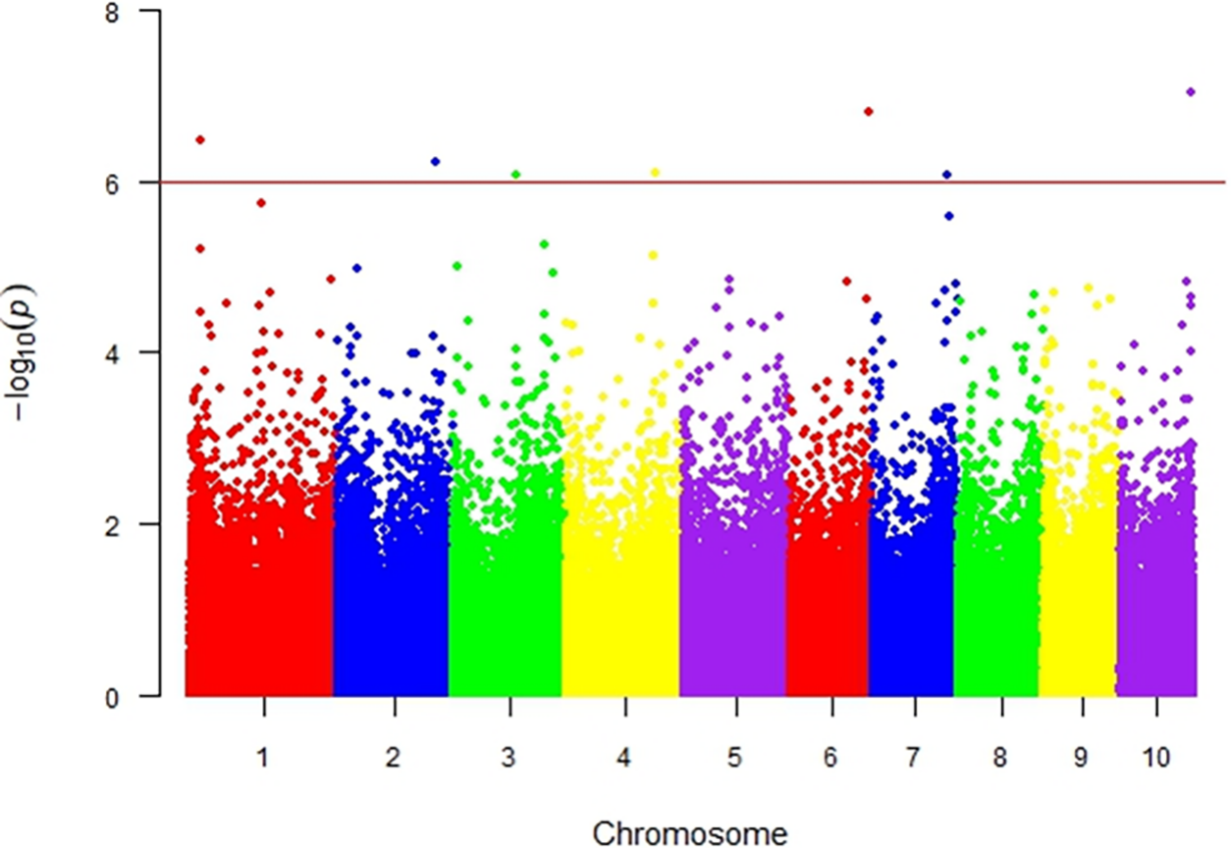
Manhattan plot of p-values for marker-trait association analysis for percentage of leaf area infected by gray leaf spot. SNPs markers above the threshold line were considered significant at false discovery rate (FDR) of 5%.

In the present study, the significant SNPs were found in the chromosome bins 1.02, 2.07, 3.05, 4.08, 6.07, 7.03 and 10.07. The identified SNPs had minor allele frequencies above 5%, indicating that they were not rare and could be used in GWAS. These SNPs and their p-values are shown in the Manhattan plots (Fig 5). [29] reported a QTL at bin 7.03; Berger et al. (2014) reported QTLs at bins 4.08, 6.07, 7.03 and 10.07; [30] also found a QTL at bin 3.05, which explained 7.8% of the genetic variation. [12] found a “QTL consensus” at bin 4.08 among different studies that used recombinant mapping.

Considering GWAS, which allow the use of larger genetic backgrounds and superior numbers of molecular markers, [18] found significant SNPs at bins 2.07, 3.05, 4.08 and 7.03. [31] also found a QTL with a negative effect on GLS at bin 1.02 using association mapping. It is apparent from all the previous studies that GLS resistance is influenced by multiple genes with medium to small effects, with few consensuses between significantly associated bins among different studies.

Version 3 of the B73 inbred line (RefGen_v3) available at MaizeGDB [32] was used to identify gene models that include or are close to the top significant SNPs. A limit of 250 kb around each SNP was checked for related genes involved in the resistance to GLS. Three different SNPs were found inside genes, and the other four SNPs were found near genes models.

## Discussion

Estimation of population structure and within-group relatedness, in maize genomic association studies, must be included in order to reduce the risk of false-positives, especially when a small number of genotypes are involved [13]. The two main PCA eigenvectors explained 13% and 3% of the variance, forming two main groups related with popcorn and field corn inbred lines, and with a mixed group between them (Fig 2). The kinship heatmap also corresponds with the popcorn and field corn groups (two main clusters), and with the different genetic relationship inside each group (Fig 3). Over fitted models for controlling spurious associations due to population structure might be a problem in GWAS analysis [33], with the risk of missing significant SNPs associated with the trait. The QQ-plot revealed a good fitting for the model with PCA and kinship, with deviations at the top of the null hypothesis diagonal, exposing a great association between the markers and the trait.

Among the seven significant SNPs found in this study, six SNPs were located inside, close (< 250 kpb) or with LD information with gene models responsible for protein synthesis and RNA polymerase subunits. Only one significant SNP was located on a gene model with unknown function. Three of these significant SNPs are related with functions pertinent to gray leaf spot resistance.

**Fig 6:**
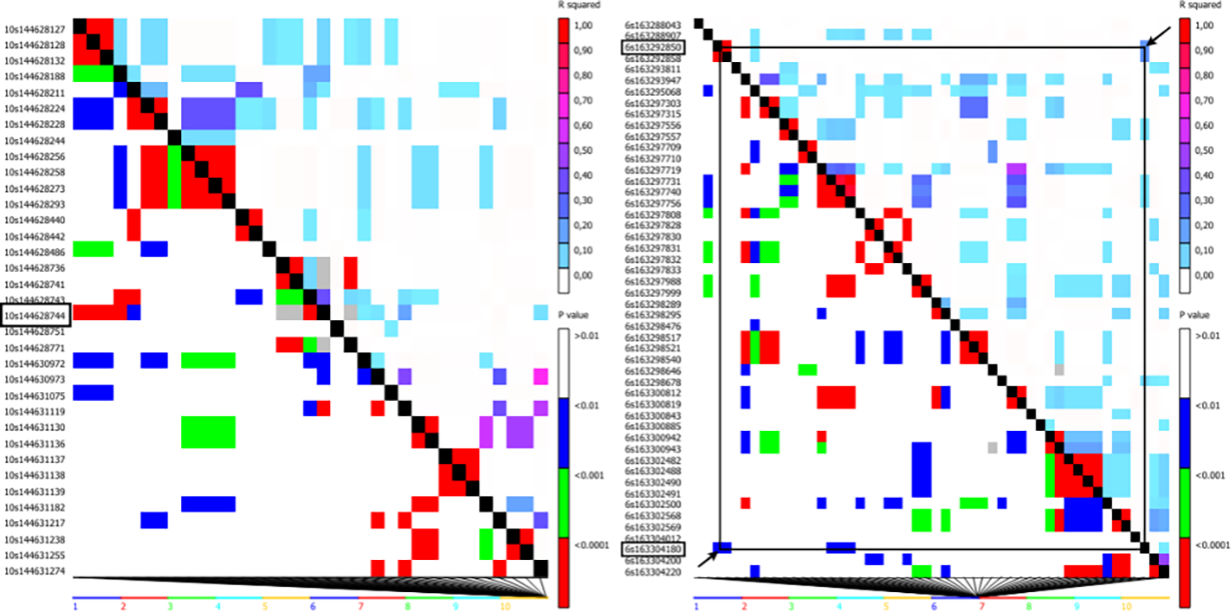
**LD heatmaps, showing the r2 measure and the p-value between the significant SNP and the SNP nearby or inside the gene model, associated with GLS resistance**. a) no LD info between SNP S1_152600619 and the gene model GRMZM2G073465 (Chr10: 185,948,327 …185,950,995); b) SNP S1_477946487 with LD info of r2 = 0.30 and p-value = 0.01 with the SNP S1_477935157 (position 163292850), inside the gene model GRMZM2G039385 (Chr6: 163,287,273 …163,296,169).

The SNP S1_152600619 is close (243.4 kb) to the functional gene model GRMZM2G073465 (bin 10.07), but no LD was found between this SNP and any SNPs near the gene (S1 Table and Fig 6). However, this gene model is associated with the recognized loci *ccp3* and encodes a protein known as cysteine protease 3. This gene model is related to a smut disease in maize caused by *Ustilago maydis*, a biotrophic pathogen that colonizes maize ears and causes significant yield losses. According to [34], the transcriptome profile indicated higher expression of this gene when maize ears were infected; however, when the cysteine protease 3 expression was silenced, the authors observed high expression of a defense gene and reduced fungal colonization. Besides being related with ear disease, the *ccp3* gene expression is also present in leaves after the flowering period [35,36,37], and the positive effect of the SNP indicates that this gene may be related with higher GLS infection.

The SNP S2_22772409 is inside to the gene model GRMZM2G007188 (bin 1.02), which encodes protein 8B from the ADP-ribosylation factor-like gene (ARF). Although this gene model has not been elucidated its function in maize metabolism pathway, RNA-Seq results have demonstrated its expression as 107.84 FPKM in leaves at eighteen days after pollination [36]. This expression is associated with the period in which the disease has higher infection rates in maize, and the negative SNP effect of -9.18 (S1 Table) may suggest a novel role of this gene model in the resistance to gray leaf spot. In plants, the ARFs are related to mitosis, cell cycle control and specially to programmed cell death for plant disease responses in order to restrict pathogen growth [38]. Using an F_2_ rice population, [39] mapped a gene that encodes a putative protein for ARF that plays an important role in the resistance to bacterial blight caused by *Xanthomonas oryzae* pv. *oryzae*.

**Fig 7:**
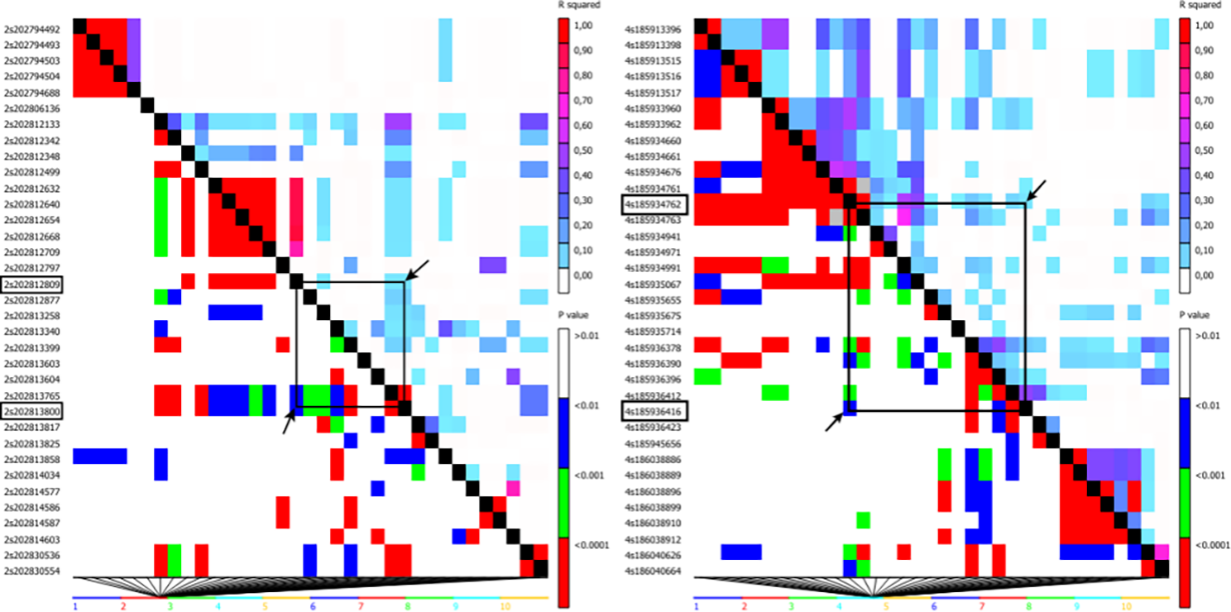
**LD heatmaps, showing the r2 measure and the p-value between the significant SNP and the SNP nearby or inside the gene model, associated with GLS resistance**: c) SNP S1_1489272307 with LD info of r2 = 0.10 and p-value = 0.01 with the SNP S1_1489273315 (position 202813817), nearby the gene model GRMZM2G154864 (Chr2: 202,829,625 …202,834,510); d) SNP S1_1710311828 with LD info of r2 = 0.10 and p-value = 0.01 with the SNP S1_1710313482 (position 185936416), nearby the gene model GRMZM2G476902 (Chr4: 185,948,327 …185,950,995).

The SNP S1_1710311828 is close (13.5 kb) to the gene model GRMZM2G476902 (bin 4.08), which encodes a putative armadillo repeat-containing protein (ARM). This significant SNP is located in a region in LD (*r*^*2*^ = 0.10; p-value = 0.01) with the closest SNP to this gene model (S1 Table, Fig 7). Proteins with the ARM repeats are related to novel functions in plants, suggesting the participation of these proteins in hormonal signaling, nuclear transport, protein degradation and response to biotic and abiotic stress [40]. In rice, mutant gene expression of *Spl11* is related to resistance to rice blast disease caused by *Magnaporthe grisea* and bacterial blight caused by *Xanthomonas oryzae* pv. *oryzae* [41]. This gene model encodes a novel protein composed of six ARM repeat domains and a U-box domain, which controls the programmed cell death caused by fungal and bacterial infections [42]. According to RNA-Seq results presented in MaizeGDB, the gene model GRMZM2G476902 has the highest expression of 20.85 FPKM in leaves 24 days after flowering [36], which is associated with the time and tissue of GLS infection. Although this gene model has not been well explained, these results and the negative SNP effect of -12.60 (S1 Table) associated with this gene model may suggest an important role in maize disease resistance.

Gray leaf spot resistance is controlled by several genes that explain a relative proportion of the phenotypic variation, which represents a major difficulty in the selection of a superior phenotype using maker-assisted selection or gene introgression. However, the development of molecular markers closely linked to functional genes is an important step to obtain improved genotypes with an acceptable degree of resistance by gene pyramiding [18]. Identification and validation of a few resistant alleles that confer a large effect on a phenotype may represent a great advance for marker-assisted selection in breeding programs.

In this study, significant SNPs were identified using the methodology of GWAS and are closely linked with candidate gene models for GLS resistance. The gene models GRMZM2G073465, GRMZM2G073465 and GRMZM2G476902 were expressed at times and in tissue that correspond to the disease infection and are good candidates for the signaling of the plant defense pathway, providing an important reference for marker-assisted selection. Other putative genes may play an important role in the process of plant defense, but it is necessary to test them in marker-assisted selection to validate their effect to gray leaf spot resistance.

## Conclusions

Seven SNPs were significant related with the maize reaction to gray leaf spot severity, and were located inside or nearby possible expressed genes. The genes models GRMZM2G073465 (bin 10.07), GRMZM2G073465 (bin 1.02) and GRMZM2G476902 (bin 4.08) may be useful for developing genotypes with resistance for gray leaf spot, through the use in molecular marker assisted selection.

## Supporting information

S1 Table SNPs significantly associated with gray leaf spot resistance at Maringá –PR. **Chromosome locations (AGPv3 coordinates), SNP position, R2, false discovery rate (FDR), genes containing SNP, allele effect and other summary statistics.** ^a^: The physical position based on B73 reference genome v3 (B73 AGPv3 bp; ^Gene containing or Adjacent to SNP; R^2^: percentage of genotypic variance explained by top significant SNPs; *Significant SNP-trait associations at false discovery rate (FDR) of 5%.
(DOCX)

S1 Appendix Gray leaf spot phenotypic data means used in in the GWAS analysis. ^1^NaN: Not a number, code used in R software, in order to substitute the values of the inbred lines which were genotyped but not phenotyped for gray leaf spot.
(DOCX)

S2 Appendix Genotype file with ~355k SNPs used in the GWAS analysis. (RAR)
