# Supplementary material for: Genome wide association study for gray leaf spot resistance in tropical maize core
Source: PLoS One. 2018 Jun 28;13(6):e0199539. doi: 10.1371/journal.pone.0199539 (PMC6023161; doi:10.1371/journal.pone.0199539)
Supplement: S1 Table — Chromosome locations (AGPv3 coordinates), SNP position, R2, false discovery rate (FDR), genes containing SNP, allele effect and other summary statistics. a: The physical position based on B73 reference genome v3 (B73 AGPv3 bp; ^Gene containing or Adjacent to SNP; R2: percentage of genotypic variance explained by top significant SNPs; *Significant SNP-trait associations at false discovery rate (FDR) of 5%. (DOCX) [file pone.0199539.s001.docx]

| SNP | Bin^a^ | Position | R^2^ | FDR | Gene^^^ | Distance | Linkage Disequilibrium (LD) | Function | Effect |
| --- | --- | --- | --- | --- | --- | --- | --- | --- | --- |
| S1_152600619 | 10.07 | 144628744 | 6.67 | 0.0048^*^ | GRMZM2G073465 | 243.4 kpb | No LD information | ccp3 - cysteine protease3 | 11.1809 |
| S1_477946487 | 6.07 | 163304180 | 7.32 | 0.0048^*^ | GRMZM2G039385 | 8.01 kpb | *r*^2^ 0.30 window, p<0.01 | Unknown | 13.1944 |
| S2_22772409 | 1.02 | 22772409 | 33.04 | 0.0048^*^ | GRMZM2G007188 | Inside | - | ADP-rybosylation factor-like protein 8B | -9.1800 |
| S1_1489272307 | 2.07 | 202812809 | 37.50 | 0.0157^*^ | GRMZM2G154864 | 1.68 kpb | *r*^2^ 0.10 window, p<0.01 | WD repeat-containing protein 74 | 5.7776 |
| S1_1710311828 | 4.08 | 185934762 | 8.53 | 0.0089^*^ | GRMZM2G476902 | 13.5 kpb | *r*^2^ 0.10 window, p<0.01 | Putative Armadillo Repeat protein | -12.5951 |
| S1_1183323714 | 3.05 | 129109843 | 1.48 | 0.0089^*^ | GRMZM2G046061 | Inside | - | DNA-directed RNA polymerase subunit | -17.2537 |
| S1_815430981 | 7.03 | 156003146 | 5.42 | 0.0089^*^ | GRMZM2G173910 | Inside | - | Putative proteasome inhibitor | 9.8171 |
