# Supplementary material for: Genome wide association study for gray leaf spot resistance in tropical maize core
Source: PLoS One. 2018 Jun 28;13(6):e0199539. doi: 10.1371/journal.pone.0199539 (PMC6023161; doi:10.1371/journal.pone.0199539)
Supplement: S1 Appendix — 1NaN: Not a number, code used in R software, in order to substitute the values of the inbred lines which were genotyped but not phenotyped for gray leaf spot. (DOCX) [file pone.0199539.s002.docx]

| **Inbred Lines** | **GLS** | **Inbred Lines** | **GLS** | **Inbred Lines** | **GLS** | **Inbred Lines** | **GLS** |
| --- | --- | --- | --- | --- | --- | --- | --- |
| 100-P7-2-3 | 10.1 | 147-P8-1-5-5 | 0.6 | 191-A11545-27 | NaN^1^ | 42-P18 | 5.35 |
| 101-AG6018-24H12.2 | 0.1 | 148-P9-8-1 | 0.6 | 192-DAS2C595-95 | NaN | 43-DKB350-76H30.1 | 0.6 |
| PREMIUM-28H13.2 | 0.6 | 149-TORK-55H20.3 | 0.6 | 193-DAS2C599-93 | NaN | 46-P4-4 | 0.6 |
| 103-CML19 | 5.35 | 14-P8-1-1 | 5.35 | 195-BEIJAFLOR-L55 | NaN | 47-P8-2-2-2 | 5.35 |
| 104-P1-3 | 20.1 | 150-P7-2-1 | 0.6 | 197-Seo13-289-2 | NaN | 48-P12-1 | 20.1 |
| 105-DKB747-45H17.5 | 0.6 | 151-FORT-84H6.1 | 0.6 | 198-RS20-257-2 | NaN | 49-AVANT-12H5.3 | 0.6 |
| 106-ANGELA-L70 | 0.6 | 152-P8-1-5-13 | 5.35 | 199-PRO23-245-1 | NaN | 4-AG8080-7H3.1 | 5.35 |
| 107-GP11-1 | 5.35 | 153-CD303-91.H4.4 | 10.1 | 19-P3-3T | 0.6 | 50-P9-7-2 | 0.6 |
| 108-FORT-85H6.2-242 | 0.6 | 154-GP3 | 0.1 | 1-GP1 | 5.35 | 51-P8-2-MULT | 20.1 |
| 109-DKB747-29H17.3 | 0.1 | DAS2C599-95H34.4 | 10.1 | 200-URUG298-98-2 | NaN | 52-DKB747-38H17.2 | 5.35 |
| 10-POP101-201-3 | 0.1 | 156-CML12 | 0.6 | PA170ROXO-324-2 | NaN | 53-P11-2 | 10.1 |
| 110-ANGELA-L65 | 0.6 | 157-GP12 | 20.1 | 202-BOYA462-110-2 | NaN | 54-GP10 | 5.35 |
| 111-AG8080-8H3.2-6 | 5.35 | 159-GP5 | 10.1 | 203-BOZM-260-36-2 | NaN | 56-DKB747-36H17.2 | 0.6 |
| 112-VICOSA-L88 | 0.6 | 15-P20 | 5.35 | 205-BaraoVicosa-134-2 | NaN | 57-P7-4-11 | 5.35 |
| 115-DKB747-37H17.2 | 5.35 | 160-SPEED-81H33.1 | 0.1 | 206-CHZM13134-66-2 | NaN | 58-P9-5-1 | 5.35 |
| 116-BEIJAFLOR-L59 | NaN | 161-P6-11 | 0.6 | 207-SAM274-2 | NaN | 59-POP202-177.1 | 0.6 |
| 117-GP14 | 0.6 | 162-P1780 | 0.6 | 209-PARA172-76-2 | NaN | 5-T5-AVANT-14H5.5 | 5.35 |
| 119-VICOSA-L77 | 0.6 | 163-POP102-91.2 | 0.6 | 20-T1-P8-2 | 20.1 | 60-FLASH-20H11.1 | 0.6 |
| 11-DKB350-78H30.1 | 0.1 | 164-30-11 | 0.6 | 210-ARZM0583-122-2 | NaN | 61-DAS422-80H31.2 | NaN |
| 120-P15 | 10.1 | 165-POP101-197.1 | 0.6 | 212-CMS | NaN | 62-P8-2-2-5 | 10.1 |
| 121-ANGELA-L71 | 5.35 | 167-29-154 | 0.1 | 21-STRIKE-67H25.1 | 0.6 | 63-AG9090-56H21.1 | NaN |
| 122-30F33-70H23.1 | 0.6 | 169-DAS422-79H31.1 | 0.6 | 22-ANGELA-L63 | NaN | 64-P8-1-5-10 | 5.35 |
| 124-UFV-L80 | 5.35 | 16-FORT-87H6.4-248 | 0.6 | 23-P8-1-5-4 | NaN | 65-P9-1-2 | 5.35 |
| 126-30F98-75H29.2 | NaN | 170-DKB747-40H17.3 | 0.6 | 24-POP201-195.1 | NaN | 66-30-23 | 20.1 |
| 127-ANGELA-L66 | 20.1 | 171-FORT-86H6.3 | 0.6 | 25-30F33-69H26.1 | 5.35 | 67-POP201-198.4 | 0.6 |
| 128-P3-1-2 | 0.6 | 173-A2560-170 | NaN | 26-P1-12 | 0.6 | 68-A2560-66H23.4 | 0.1 |
| 129-P1-8 | 0.6 | 174-A2560-176 | NaN | 27-GP13 | 0.6 | 69-TORK-54H20.3 | 10.1 |
| 12-DKB747-50H17.6 | 0.6 | 175-A2560-164 | NaN | 28-P9-1 | 10.1 | 6-POP103-88.1 | 0.6 |
| 130-BEIJAFLOR-L52 | 40.1 | 176-DKB747-41-101 | 0.1 | 29-P7-2-4 | 5.35 | 70-A2560-64H23.2 | NaN |
| 131-CD303-90H4.3 | NaN | 177-DKB747-47-121 | 0.6 | 2-GP4 | 0.6 | 71-31-88 | NaN |
| 134-P9-5-3 | NaN | 178-DKB747-48-124 | 0.6 | 30-DKB747-43H17.4 | 0.6 | 72-P7-4-5 | 20.1 |
| 136-TORK-53H20.2 | 0.6 | 179-DAS422-8-222 | 0.1 | 31-CD303-89H4.2 | 10.1 | 7-30F33-71H26.2 | 0.1 |
| 137-POP203-56.1 | 5.35 | 17-P1-9 | 20.1 | 32-30F98-74H29.1 | NaN | 74-P9-12-1 | 0.1 |
| 138-POP102-166.5 | 0.6 | 180-DKB747-42-104 | 5.35 | 33-P9-4-5 | 5.35 | 76-POP102-90.1 | 0.6 |
| 139-A2560-62H23.2 | 0.1 | 181-AG9090-57-155 | 0.1 | 35-VICOSA-L75 | 30.1 | 77-29-14 | 0.1 |
| 13-BEIJAFLOR-L53 | 10.1 | 182-DKB747-44-110 | 10.1 | 36-P6-1 | 10.1 | 78-31-97 | NaN |
| 140-POP101-195.2 | 0.6 | 183-P9-2-3 | 5.35 | 37-GP15 | 5.35 | 79-POP202-88.2 | 0.6 |
| 141-P9-1-3 | 5.35 | 185-P9-1-6 | 10.1 | 38-AVANT-10H5.1 | 0.6 | 80-30-15 | NaN |
| 142-FLASH-22H11.1 | 0.6 | 188-DKB350-77-H30.1 | NaN | 39-DKB747-41H17.3 | 0.6 | 81-T3-P9-3-2 | NaN |
| 144-P9-11-1 | 10.1 | 189-DKB440-73-H28.1 | NaN | 3-P9-4-6 | 10.1 | 82-POP201-192.1 | 0.1 |
| 145-BEIJAFLOR-L76 | 0.6 | 18-P11-1 | 20.1 | 40-P8-1-5-9 | 10.1 | 83-29-92 | 0.1 |
| 146-A2560-63H23.2 | 0.6 | 190-Premium-29-h13.3 | NaN | 41-P1-19 | 10.1 | 84-POP203-51.2 | 0.1 |
| 85-29-174 | NaN | 89-30-29 | NaN | 91-31-124 | NaN | 94-P8-2-2-4 | 0.6 |
| 86-CML13 | 5.35 | 8-P7-L7-1 | 5.35 | 92-POP202-76.1 | 0.1 | 95-CML22 | 0.6 |
| 88-POP103-80.5 | 0.1 | 90-POP103-81.4 | 0.6 | 93-31-33 | 0.6 | 96-P19 | 10.1 |
| 97-DKB350-19H9.1 | 0.1 | 98-AVANT-13H5.4 | 5.35 | 99-W57 | NaN | - | - |
